# Involvement of Aquaporin 3 in *Helicobacter pylori*-Related Gastric Diseases

**DOI:** 10.1371/journal.pone.0049104

**Published:** 2012-11-09

**Authors:** Gang Wang, Fei Gao, Weiming Zhang, Jia Chen, Tao Wang, Guoxin Zhang, Lizong Shen

**Affiliations:** 1 Division of Gastrointestinal Surgery, Department of General Surgery, First Affiliated Hospital, Nanjing Medical University, Nanjing, Jiangsu, China; 2 Department of Pathology, First Affiliated Hospital, Nanjing Medical University, Nanjing, Jiangsu, China; 3 Department of Gastroenterology, First Affiliated Hospital, Nanjing Medical University, Nanjing, Jiangsu, China; Veterans Affairs Medical Center (111D), United States of America

## Abstract

**Background:**

Gastric cancer is one of the most common and lethal malignant cancers worldwide, and numerous epidemiological studies have demonstrated that *Helicobacter pylori* (*H. pylori*) infection plays a key role in the development of gastric carcinomas. Our previous studies showed that aquaporin 3 (AQP3) is overexpressed in gastric carcinoma and promotes the migration and proliferation of human gastric carcinoma cells, suggesting that AQP3 may be a potentially important determinant of gastric carcinoma. However, the role of AQP3 in *H. pylori* carcinogenesis is unknown.

**Methods:**

The AQP3 protein and *H. pylori* were detected in human gastric tissues by immunohistochemistry and modified Giemsa staining respectively. AQP3 knockdown was obtained by small interfering (si) RNA. Western blot assays and RT-PCR were used to evaluate the change of AQP3 in the human gastric cancer AGS and SGC7901 cell lines after co-culture with *H. pylori*. Sprague Dawley rats were orally inoculated with *H. pylori* to establish a rat model colonized by *H. pylori*.

**Results:**

The present study found that AQP3 expression correlated with *H. pylori* infection status in gastric cancer tissues and corresponding normal mucosa, and *H. pylori* co-culture upregulated AQP3 expression in human gastric adenocarcinoma cells *in vitro* via the extracellular signal-regulated kinase signaling pathway. *H. pylori* infection also increased AQP3 expression in gastric mucosa colonized by *H. pylori* in a Sprague Dawley rat model.

**Conclusions:**

These findings provide further information to understand the mechanism of *H. pylori* carcinogenesis and a potential strategy for the treatment of *H. pylori*-associated gastric carcinoma.

## Introduction

Gastric carcinoma remains one of the most common and lethal malignancies worldwide, with approximately 1 million cases diagnosed annually, accounting for 738,000 deaths in 2008. Over 70% of the new cases and deaths occur in developing countries [1]. Numerous epidemiological studies have indicated that *Helicobacter pylori* (*H. pylori*) plays a key role in the development of both intestinal-type and diffuse-type gastric carcinomas, especially in the distal portion of the stomach [2]–[4]. Regional variations in the incidence partially reflect differences in the prevalence of *H. pylori* infection [5], [6]. Stomach cancer rates have decreased substantially in most parts of the world [7], partially because of reductions of chronic *H. pylori* infection in these areas [8]–[10]. *H. pylori* has been demonstrated to promote tumorigenesis [11]. *H. pylori* infection is defined as a definite or Class I carcinogen in the human stomach by the World Health Organization [3]. *H. pylori* strains produce various toxins that enable the bacteria to cause host cell damage, including cytotoxin-associated gene A (CagA) and vacuolating cytotoxin (VacA) [12]. Recent studies showed that infection with CagA-positive *H. pylori* plays an essential role in the development of gastric carcinoma. The cagA-encoded CagA protein is delivered into gastric epithelial cells via the bacterial type IV secretion system [2]. Moreover, CagA interacts with many signaling molecules and elicits a series of cellular events. Changes related to cell morphology, cell scattering, cell proliferation, and intercellular tight junctions have also been identified [13]. *H. pylori* infection may cause a combination of increased endogenous DNA damage, decreased repair activity, and the induction of mutations in mitochondrial DNA that generate genetic instability in gastric cells and promote gastric carcinogenesis [14].

Aquaporins (AQPs) are a family of small, integral membrane proteins that transport water and, in some cases, water and glycerol (“aquaglyceroporins”) [15], [16]. Aquaporins are involved in transepithelial fluid transport, as it occurs in the urinary concentrating mechanism and glandular fluid secretion. Accumulating evidence further implicates AQPs in cell migration and proliferation, adding AQPs to an expanding list of effectors in tumor biology [17]. We showed previously that human gastric carcinoma tissues expressed higher levels of AQP3 than normal mucosa, and AQP3 expression was associated with histological classification, lymph node metastasis, and lymphovascular invasion [18], [19], suggesting that AQP3 may play an important role in human gastric cancer. In addition, we showed that AQP3 promotes the migration and proliferation of human gastric carcinoma AGS and SGC7901 cells, suggesting that AQP3 may be a potentially important determinant of tumor growth and the spread of human gastric carcinoma [20], [21].

However, the role of AQP3 in *H. pylori* carcinogenesis remains unclear. Based on our previous findings, we speculated that AQP3 may play an important role in *H. pylori*-related gastric diseases. In the present study, we investigated the relationship between the expression of AQP3 in gastric carcinoma tissues or corresponding normal mucosa and *H. pylori* infection status, and the effects of *H. pylori* on AQP3 expression in gastric cells were examined *in vitro* and *in vivo*. We found that AQP3 expression in gastric cancer tissues correlated with *H. pylori* infection status, and *H. pylori* upregulated AQP3 expression in human gastric adenocarcinoma cells *in vitro* via the extracellular signal-regulated kinase (ERK) signaling pathway, confirmed by experimental gastric helicobacter infection in rats. These findings provide further information to understand the mechanism of *H. pylori* carcinogenesis and a potential strategy for the treatment of *H. pylori*-associated gastric carcinoma.

## Materials and Methods

### Human Gastric Tissue Specimens

Human gastric tumor tissue specimens were randomly obtained from 89 gastric adenocarcinoma patients (median age, 56 years; range, 35–75 years; 58 males, 31 females) between June 2007 and September 2008 at the Department of General Surgery, First Affiliated Hospital, Nanjing Medical University, Nanjing, China. All of the patients were diagnosed pathologically according to the American Joint Committee on Cancer criteria. Samples of tumor and corresponding non-cancerous tissue from all of the patients were collected immediately after resection and snap-frozen in liquid nitrogen. Based on the Lauren classification, 54 cases were intestinal cancer, and 35 cases were diffuse gastric cancer. The tumors of 15, 26, and 48 cases were located in the upper third, middle third, and lower third of the stomach, respectively. Thirteen cases were categorized as stage I, 19 cases were stage II, and 57 cases were stage III. No case with distant metastasis was found in this study. The acquisition of tissue specimens and study protocol were approved by the Nanjing Medical University Institutional Review Board, and the study was performed in strict accordance with the regulations. Written informed consent was obtained from all of the patients.

### Immunohistochemistry (IHC) for AQP3 Protein Expression in Specimens

The expression of AQP3 in the specimens was determined by IHC as described previously [21]. Anti-AQP3 antibody (Santa Cruz Biotechnology, Santa Cruz, CA) was used. A pathologist coded AQP3 expression as the percentage of positive tumor cells (scale 0%∼100%) with staining intensity from 0 to 3+. Positive IHC expression is defined as 25% or more staining with intensity 2 or 3+.

### Modified Giemsa Staining of *H. pylori* in Gastric Mucous Tissues

Modified Giemsa staining was used to detect *H. pylori* in gastric mucous tissues according to a previous report [22].

### Cells and RNA Interference (RNAi)

The human gastric cancer AGS (ATCC, Manassas, VA) and SGC7901 (CBTCCCAS, Shanghai, China) cell lines were cultured in RMPI-1640 (Life Technologies, Gibco BRL, Grand Island, NY, USA) supplemented with 10% fetal bovine serum (FBS; Invitrogen), penicillin/streptomycin (1∶100; Sigma, St. Louis, MO), and 4 mM glutamine (Life Technologies, Gibco BRL) in a humidified atmosphere that contained 5% CO_2_ at 37°C. RNAi assays were performed according to a previous report [21].

### *H. pylori* Culture and Co-culture with Gastric Cells

Experiments were performed with a cytotoxic (CagA+ and VacA+) reference strain of *H. pylori* 26695 (ATCC). *H. pylori* bacteria were grown under microaerophilic conditions on Columbia agar plates (bioMérieux, Marcy l'Etoile, France) that contained 100 U/ml *H. pylori* selective supplement (Oxoid, Basingstoke, United Kingdom) at 37°C using an anaerobic chamber (BBL Campy Pouch System, Becton Dickinson Microbiology Systems) for 48–72 h, harvested, and resuspended in antibiotic-free RPMI 1640 medium (Life Technologies, Gibco BRL) supplemented with 2% fetal calf serum (FCS). The bacterial densities were adjusted by optical density (OD) measurement at 660 nm, in which 1 OD_660_ = 1×10^8^ colony-forming units (CFU)/ml. *H. pylori* was then incubated with AGS or SGC7901 cells at a cell-to-bacterium ratio of 1∶50 or 1∶100 for up to 12 or 24 h in the medium.

### Western Blot Assays

Cellular or tissue extracts were prepared with ice-cold radioimmunoprecipitation assay buffer that contained 50 mM Tris-HCl (pH 7.4), 150 mM NaCl, 1% NP-40, 1 mM ethylenediaminetetraacetic acid, 0.25% sodium deoxycholate, 1 mM NaF, 10 µM Na_3_VO_4_, 1 mM phenylmethanesulfonyl fluoride, and a cocktail of protease inhibitors (10 µg/ml leupeptin, 10 µg/ml aprotinin, and 1 µM pepstatin). The concentrations were determined by the bicinchoninic acid protein assay. Immunoblotting was performed as previously described [21], and the following antibodies were used for the procedure: anti-AQP3 antibody (Santa Cruz Biotechnology, Santa Cruz, CA), anti-phospho-ERK and anti-ERK antibody (Cell Signaling Technology, Beverly, MA), and anti-β-actin antibody (Santa Cruz Biotechnology). Protein bands were detected using an enhanced chemiluminescence (ECL) detection system according to the manufacturer’s instructions and visualized by autoradiography with Hyperfilm. Protein densitometric analysis was performed with normalization against β-actin.

### Real-time Quantitative Polymerase Chain Reaction (RT-qPCR) [23]

Total RNA from the cells was prepared with Trizol (Invitrogen, Carlsbad, CA) according to the manufacturer’s protocol under RNase-free condition. After cDNA was synthesized with a two-step reverse transcription reaction kit (TAKARA, Dalian, China), quantitative PCR was carried out on an Applied Biosystems 7500 Real-time PCR System using SYBR Premix Ex Taq Kit (Applied Biosystems, USA) in Axygen 96-well reaction plates, following the manufacturer’s instructions. β-actin was amplified to serve as a reference for each sample and the observed AQP3 expression level was normalized to the level of β-actin. The following pairs of primers were AQP3, forward primer corresponding to nucleotides 5′- CACAGCCGGCATCTTTGCTA-3′ and reverse primer complimentary to nucleotides 5′-TGGCCAGCACACACACGATA-3′; β-actin, forward primer 5′- TCACCCACACTGTGCCCATCTACGA-3′ and reverse primer 5′- CAGCGGAACCGCTCATTGCCAATGG -3′. All procedures were performed in triplicate.

### *In vitro* Migration (scratch) Assay and Cell Proliferation Assay

In vitro migration was assayed as previously described [20]. The representative images of the scratched areas under each condition were photographed. To estimate the relative migration of the cells, the unclosed cell-free areas from five prints under each condition were excised. Migration was quantified as the “average gap” (average gap, the percentage of the unclosed cell-free areas according to initial scratch distance). The polylysine alone at 0 h was considered 100% of the average gap. For the cell migration experiment, at least 50 cell migration distances were counted for one experiment. Cell proliferation was analyzed using a WST-8 Cell Counting Kit-8 (CCK-8; Sigma) according to the manufacturer’s protocol. The results were plotted as mean ± standard error (SE) of three separate experiments having four determinations per experiment for each experimental condition.

### Establishment of a Sprague Dawley Rat Model Colonized by *H. pylori*

Thirty Sprague Dawley rats (Vitalriver, Nanjing, China; weight, 180±20 g) were randomly divided into two groups: *H. pylori* treatment group and control group, each containing fifteen rats. Rats in the *H. pylori* treatment group were orally inoculated with *H. pylori* 26695 resuspended in 4 ml phosphate-buffered saline (PBS) that contained 5×10^8^ CFU/ml. *H. pylori* treatment was performed twice per week and lasted 4 consecutive weeks. Before inoculation, these rats were fasted for 24 h and slowly pretreated with 3 ml of 5% NaHCO_3_ intragastrically 30 min before inoculation. The animals in the control group were administered PBS accordingly. Three months after inoculation, gastric mucous tissues in the antrum were harvested for further assay, including pathological diagnosis with hematoxylin-eosin staining using the Visual Analog Scale of the Updated Sydney System [24], modified Giemsa staining with cultivation and rapid urease test for *H. pylori* detection, and Western blot to assess AQP3 expression.

### Statistical Analysis

The data are expressed as mean ± standard error. In the experiments that involved protein expression, the values were representative of at least three independent experiments. Clinicopathological findings were compared using an unpaired *t*-test or the Pearson *χ^2^* test. The statistical analysis of the data between the control and treated groups was performed using analysis of variance (ANOVA). Values of *p*<0.05 were considered statistically significant.

## Results

### AQP3 Expression in Gastric Cancer Tissues Correlates with *H. pylori* Infection Status

**Figure 1:**
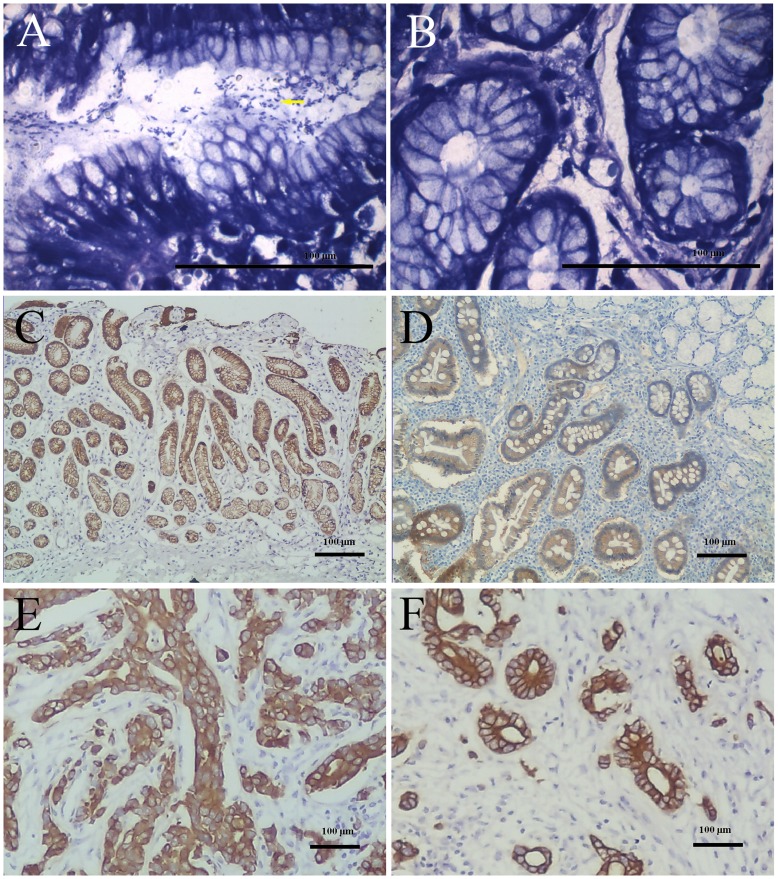
AQP3 expression and *H. pylori* detection in human gastric cancer by IHC and Giemsa staining. Strong positive (+++) AQP3 immunoreactivity was identified in moderately differentiated adenocarcinoma (E) with *H. pylori* detection in the gastric glands (A, yellow arrow), which was stronger than in the corresponding non-cancerous mucosa (+) (C). However, weak positive AQP3 reactivity (+) was observed in well-differentiated adenocarcinoma (F) with negative *H. pylori* detection (B), and AQP3 reactivity in the corresponding normal mucosa was weaker (D).

AQP3 protein expression was evaluated by IHC and *H. pylori* infection by modified Giemsa staining in 89 human gastric tumor tissue specimens and corresponding non-cancerous mucosa in the antrum. The prevalence of *H. pylori* in these patients was 59.6% (53/89; Table 1). As we reported previously, AQP3 protein expression in gastric cancer tissues was higher than in corresponding normal tissues [19], although weak to moderate positive AQP3 reactivity was found in the non-cancerous mucosa (Fig. 1). *H. pylori* colonized the lumen of the gastric glands in 51 cases (57.3%) (Fig. 1). However, AQP3 reactivity in both normal gastric mucosa and gastric cancer tissues was markedly associated with *H. pylori* infection (*p = *0.000; Table 1), indicating that AQP3 may be involved in *H. pylori* infection-related gastric diseases.

**Table 1:** AQP3 expression is associated with *H. pylori* infection status.

Variables	AQP3	*H. pylori* positive	*H. pylori* negative	X^2^	*P*
Normal gastric mucosa	±	11	24	18.94	<0.001
	+	42	12		
Gastric cancer tissues	+	8	21	18.25	0.001
	2+∼3+	45	15		

### *H. pylori* Upregulates AQP3 Expression in Human Gastric Adenocarcinoma Cells *in vitro*

**Figure 2:**
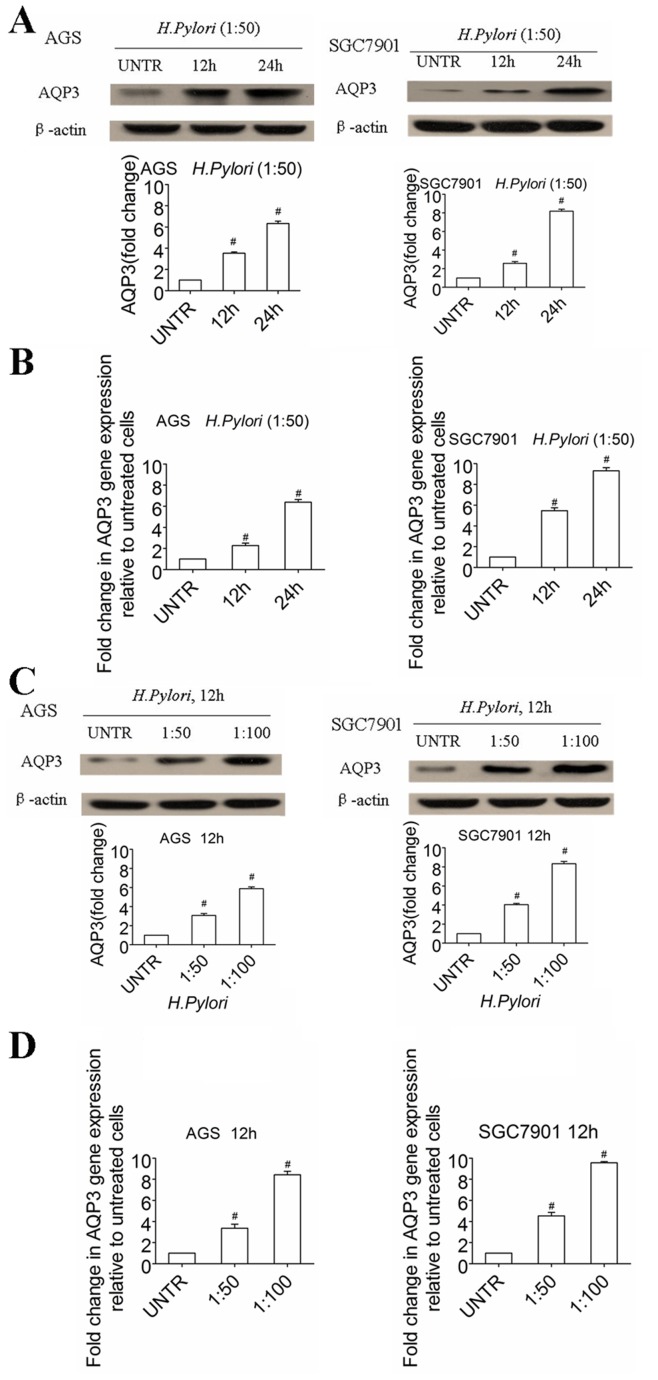
*H. pylori* treatment upregulated AQP3 expression in human gastric adenocarcinoma cells *in vitro*. Cultured AGS and SGC7901 cells were treated with *H. pylori* (1∶50). AQP3 protein and mRNA expression was analyzed by Western blot and RT-qPCR in (A) and (B) at different time points (12 and 24 h) and quantified with normalization against β-actin. The cells were also treated with various doses of *H. pylori* (1∶50, 1∶100), and AQP3 protein and mRNA expression was analyzed after 12 h and quantified as normalization against β-actin (C and D). The data are expressed as the mean ± SE of three independent experiments. ^#^ *p*<0.05, compared with untreated (UNTR) group.

*In vitro* experiments were performed to investigate the effects of *H. pylori* infection on AQP3 expression in human gastric carcinoma cells. We co-cultured SGC7901 and AGS gastric carcinoma cells with *H. pylori*. Immunoblotting assays with anti-AQP3 antibody showed that *H. pylori* infection dose- and time-dependently increased the protein levels of AQP3 in both cell lines (Fig. 2A and 2C). Furthermore, AQP3 expression increased approximately two- to three-fold in AGS cells and two- to four-fold in SGC7901 cells in the *H. pylori* co-culture over a range of 1∶50 to 1∶100 (cells to bacteria). AQP3 expression also increased over time by 1.5- to two-fold at 12 h to over three- to four-fold at 24 h posttreatment with *H. pylori* (1∶50) in both cell lines.

To investigate whether *H. pylori* upregulates AQP3 expression at the transcriptional or posttranscriptional level, RT-qPCR assays were performed to examine the mRNA transcript levels of AQP3 in AGS and SGC7901 cells. We found that *H. pylori* significantly increased the mRNA transcript levels of AQP3 in both cell lines (Fig. 2B and 2D).

### *H. pylori* Infection Induces Migration and Proliferation of Gastric Cancer Cells via AQP3

**Figure 3:**
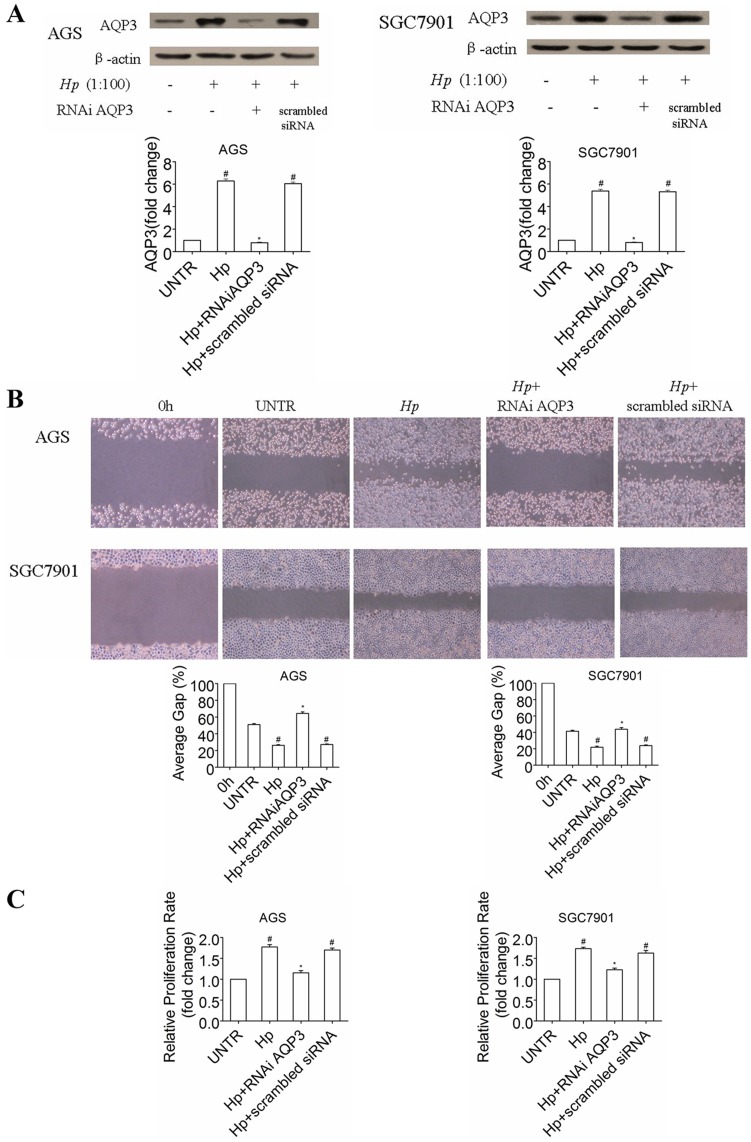
*H. pylori* treatment promotes cell migration and proliferation via AQP3 *in vitro*. AQP3 siRNA abated the level of AQP3 in AGS and SGC7901 cells (A). AGS and SGC7901 cells were treated with or without AQP3 siRNA and scrambled siRNA, followed by treatment with *H. pylori* (1∶100) for 24 h. AQP3 protein expression was analyzed by Western blot (A) with quantification normalized against β-actin or the untreated (UNTR) group. Cell migration was detected by *in vitro* migration (scratch) assays as described previously [20] and photographed at 24 h (B) to determine the average gap. Changes in cell proliferation in each group were detected by CCK-8 and are expressed as the fold change (C). The data are expressed as the mean ± SE of three independent experiments. ^#^ *p*<0.05, compared with untreated (UNTR) group. *p<0.05, compared with *H. pylori*-treated group.

As shown in many studies, *H. pylori* infection promotes the migration and proliferation of gastric epithelial cells, which is thought to be critical characteristic of *H. pylori*-associated gastric cancer [25], [26]. Our previous study [20] prompted us to investigate whether *H. pylori* regulates the promotion and migration of gastric mucosa cells via AQP3. We transfected the human gastric cancer cell lines with siRNA against AQP3 or scrambled siRNA. Immunoblotting assays showed that siRNA against AQP3 reduced AQP3 expression by approximately two-thirds in both AGS and SGC7901 cells. Additionally, AQP3 knockdown significantly attenuated the *H. pylori*-induced increase in AQP3 expression in both AGS and SGC7901 cells (Fig. 3A). Accordingly, AQP3 knockdown inhibited the *H. pylori*-induced increase in gastric cell proliferation and migration (Fig. 3B and 3C). These data suggest that AQP3 is involved in *H. pylori*-induced changes in cell behavior.

### The ERK Signaling Pathway is Involved in *H. pylori*-regulated AQP3 Expression in Human Gastric Cancer Cells

**Figure 4:**
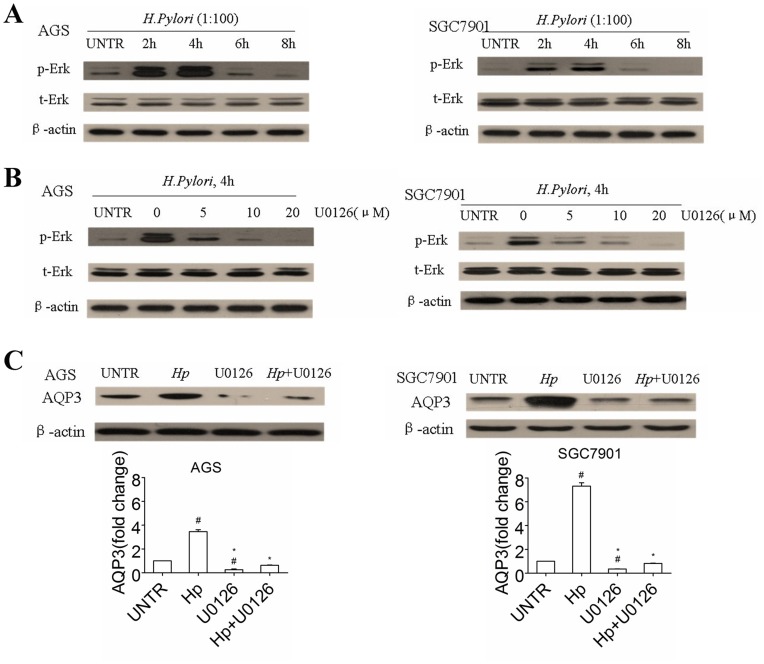
*H. pylori* treatment upregulates AQP3 expression in gastric adenocarcinoma cells via the ERK pathway. *H. pylori* treatment induced ERK activation in human gastric adenocarcinoma cells, which reached a peak at 2 h and continued at this level for approximately 4 h (A). AGS cells and SGC7901 cells were pretreated with various doses of the MAPK/ERK inhibitor U0126 (5, 10, and 20 µM) for 1.5 h, followed by *H. pylori* (1∶100) for 4 h. ERK activation (pERK) was detected by Western blot (B) to determine the optimal dose of U0126. The cells were treated with *H. pylori* (1∶100) with or without the MAPK/ERK inhibitor U0126 (10 µM) for 2 h, and AQP3 expression was analyzed by Western blot and quantified with normalization against β-actin (C). ^#^ *p*<0.05, compared with untreated (UNTR) group. *p<0.05, compared with *H. pylori*-treated group.

The activation of ERK signaling pathway is involved in cell migration and proliferation [27], and our previous study showed that AQP3 expression in gastric cells is regulated mainly via the ERK signaling pathway [20], [21]. We further investigated whether the ERK signaling pathway is involved in *H. pylori*-regulated AQP3 expression in human gastric cancer. Immunoblotting studies using anti-phospho-ERK antibody revealed the phosphorylation of ERK in both AGS and SGC7901 cells at 2 h posttreatment with *H. pylori* (1∶100), which continued at this level for approximately 4 h and returned to baseline at 8 h posttreatment (Fig. 4A), which was consistent with other report [28]. U0126, a small-molecule inhibitor of mitogen-activated protein kinase (MAPK)/ERK, abrogated *H. pylori*-induced ERK phosphorylation (Fig. 4B). Furthermore, pretreatment with 10 µM U0126 for 2 h partially inhibited the *H. pylori*-induced increase in AQP3 expression (Fig. 4C). These data indicate that the ERK signaling pathway is involved in *H. pylori*-regulated AQP3 expression in human gastric carcinoma cells.

### *H. pylori* Infection Upregulates AQP3 Expression in an Experimental Gastric Helicobacter Infection Rat Model

**Figure 5:**
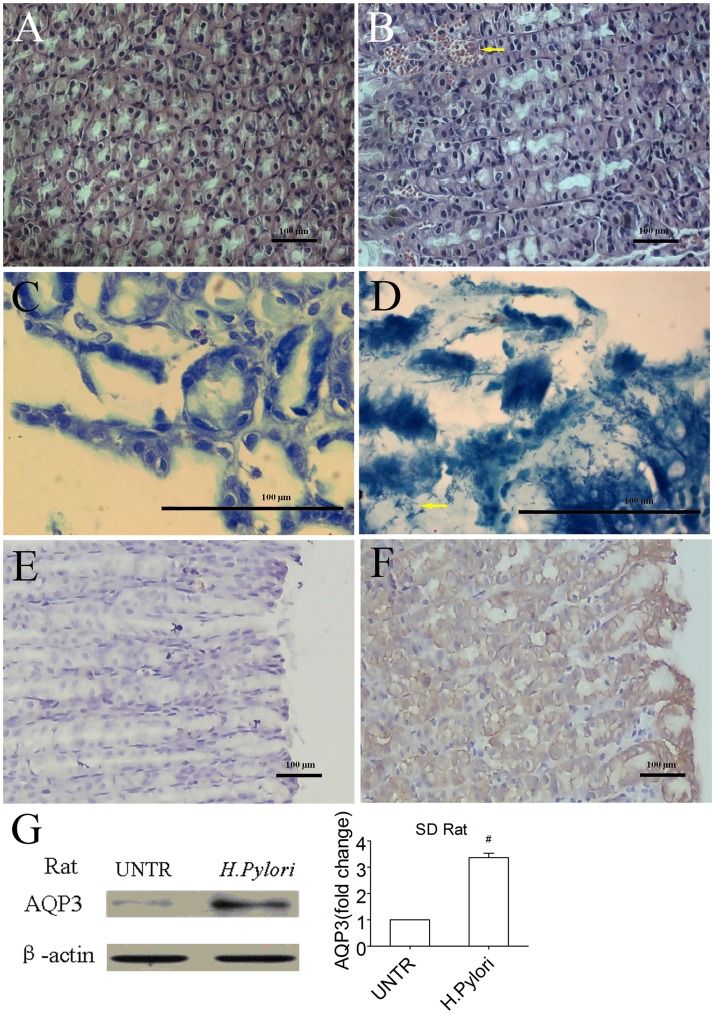
*H. pylori* infection upregulates AQP3 expression in an experimental gastric helicobacter infection model in rats. A and B represented gastric tissue sections from rats with hematoxylin-eosin staining. The gastric mucosa (B) with *H. pylori* treatment showed moderate histological findings, including submucosal bleeding (blank arrow) and mononuclear cell infiltration (small arrow). No marked histological changes were found in the mucosa from rats in the control group (A). Large numbers of bacteria (yellow arrow) were seen colonizing the lumen of the gastric glands in the *H. pylori*-treated group (D) with modified Giemsa staining, and no bacterium was found in the control group (C). Positive AQP3 immunoreactivity was identified in the gastric mucosa (F) in rats with *H. pylori* treatment. AQP3 reactivity was not observed in the gastric mucosa (E) in the control group, which was confirmed by the Western blotting assay (G).

To further investigate the role of AQP3 in *H. pylori*-related gastric diseases, a Sprague Dawley rat model colonized by *H. pylori* was established. The gastric mucosa in the rats with *H. pylori* treatment showed moderate histological findings, including submucosal bleeding and mononuclear cell infiltration. No marked histological changes were found in the mucosa in the rats in the control group (Fig. 5A and 5B). Modified Giemsa staining revealed large numbers of bacteria that colonized the lumen of the gastric glands in the *H. pylori*-treated groups, and no bacterium was found in the control groups (Fig. 5C and 5D). Furthermore, cultivation of rat gastric mucosa on Columbia agar plates showed that *H. pylori* colony formed in the *H. pylori*-treated groups and no colony in the control groups, which were confirmed by the rapid urease test after colony formation. These results indicated that the experimental gastric *H. pylori* infection model was successfully established. Importantly, AQP3 expression in the gastric mucosa of the *H. pylori*-treated group was stronger than in the control group, indicating that AQP3 is involved in *H. pylori* infection (Fig. 5E–G).

## Discussion

*H. pylori* is an important pathogen involved in gastritis, peptic ulcers, gastric carcinoma, and primary gastric lymphoma. The prevalence of *H. pylori* infection differs significantly among countries, with a higher prevalence in developing countries compared with developed countries. Shi et al. [6] reported that *H. pylori* was detected in 851 individuals among 1371 subjects (62%) in a Chinese prospective epidemiologic survey of *H. pylori* infection, and the prevalence reached a peak at an age of 30–40 years. Although *H. pylori* infection rates have decreased in the general Chinese population during recent years, the prevalence of *H. pylori* infection among both children and adults remains significantly higher in areas with a high incidence of gastric cancer in China compared with areas with a low incidence of gastric cancer [29]. Gastric carcinoma is the second most frequently diagnosed cancer and third leading cause of cancer death in China, with an estimated 464,439 new cases and 352,315 cancer deaths in 2008 [30], accounting for 42% of all gastric cancer cases worldwide.

Several recent clinical studies have reported an association between *H. pylori* infection and gastric carcinogenesis [31]–[36]. Umemura et al. [31] found that gastric cancer developed in 36 of 1246 *H. pylori*-infected patients but none of the 280 uninfected patients in a prospective study that involved 1526 Japanese patients with peptic ulcers, gastric hyperplasia, and non-ulcer dyspepsia. This seminal study offered compelling evidence that *H. pylori* infection is associated with the development of both intestinal and diffuse gastric cancers. The role of *H. pylori* in the development of gastric cancer has also been demonstrated in clinical studies conducted in Chinese. The majority of epidemiologic studies that examined the association between *H. pylori* infection and gastric cancer in China were retrospective case-control studies. All 11 case-control studies included in a 2001 meta-analysis showed a positive association [32]. A recent hospital-based case-control study conducted in Taiwan also showed that *H. pylori* infection increased gastric adenocarcinoma risk, including cardia and non-cardia [33]. This positive association was also observed in two prospective cohort studies. Yuan et al. [34] reported that the odds ratio was 3.74 for individuals who were seropositive for *H. pylori* and followed for 5 or more years in a nested case-control study with a cohort of Shanghai residents. A prospective, nested case-control study in Linxian, one of the highest-incidence regions in China, found that *H. pylori* seropositivity was associated with an approximately two-fold increased risk of gastric cancer [35]. These results were confirmed by a 2007 case-cohort study, in which *H. pylori* was associated with a 1.6-fold increased risk of both cardia and non-cardia gastric adenocarcinomas [36].

The eradication of colonizing *H. pylori* decreases the risk of developing gastric cancer. A multicenter, open-label, randomized controlled trial followed 544 patients who underwent endoscopic resection of early gastric cancer, half of whom underwent eradication of colonizing *H. pylori* [37]. Eradication decreased the risk by approximately 65%, although these patients had already been diagnosed with early gastric cancer. To determine whether *H. pylori* eradication reduces the incidence of gastric cancer at the population level in high-risk areas in China, Wong et al. [38] conducted a randomized, placebo-controlled trial in subjects without precancerous lesions. Although the difference in the incidence of gastric cancer development between participants who received *H. pylori* eradication treatment and patients who received placebo for 7.5 years in a high-risk region of China was not significant, eradication of *H. pylori* significantly decreased the development of gastric cancer in the subgroup of *H. pylori* carriers without precancerous lesions. In the Shandong Intervention Trial, 2 weeks of antibiotic treatment for *H. pylori* reduced the prevalence of precancerous gastric lesions [39]. Recently, they reported that gastric cancer was diagnosed in 3.0% of subjects who received *H. pylori* treatment and 4.6% of subjects who received placebo (odds ratio = 0.61, 95% confidence interval = 0.38–0.96, *p* = 0.032) after a 14.7-year follow-up for gastric cancer [40].

Our previous studies indicated that AQP3 plays an important role in the tumor growth and spread of human gastric carcinoma [20]. However, whether AQP3 is involved in *H. pylori* infection-related gastric cancer is unknown, and no studies of which we are aware have investigated alterations in the expression of AQP3 in *H. pylori*-infected stomach mucosa [41]. In the present study, we found that *H. pylori* infection upregulated AQP3 expression in non-cancerous mucosa and gastric cancer tissues.

To investigate the effect of *H. pylori* infection on AQP3 and the role of AQP3 in *H. pylori* infection, we performed an *in vitro* experiment using gastric carcinoma cells co-cultured with *H. pylori*. *H. pylori* infection dose- and time-dependently increased the protein levels and transcription of AQP3, indicating that AQP3 may be involved in *H. pylori* infection-related gastric diseases. *H. pylori* infection promoted the proliferation and migration of gastric epithelial cells, which may contribute to the carcinogenic process. RNAi assays in this study showed that knockdown of AQP3 attenuated the *H. pylori*-induced proliferation and migration of cancer cells. These data suggest that AQP3 is involved in *H. pylori*-induced changes in cell behavior. Our previous study found that AQP3 expression in gastric cells is regulated mainly via the ERK signaling pathway. We provide further evidence that the ERK signaling pathway is also involved in *H. pylori*-regulated AQP3 expression in human gastric carcinoma cells.

Furthermore, we established a Sprague Dawley rat model colonized by *H. pylori*. We did not induce gastric cancer in this model, but the animals treated with *H. pylori* presented moderate gastritis. AQP3 expression in the gastric mucosa of the *H. pylori* treatment group was higher than in the control group, directly indicating that AQP3 is involved in *H. pylori* infection.

In summary, although the present study is preliminary, our results demonstrated that AQP3 is involved in *H. pylori* infection-related diseases, especially *H. pylori*-related gastric carcinogenesis. However, we used CagA-positive *H. pylori*, *H. pylori* 26695, to treat gastric cancer cells and animals without using CagA-negative one as control. Therefore, further studies that investigate whether *H. pylori* infection upregulates AQP3 expression in gastric cancer cells via CagA protein during carcinogenesis are warranted. If a gastric cancer model induced by *H. pylori* is established, then the results may be more convincing. However, the present findings provide further information to understand the mechanism of *H. pylori* carcinogenesis and a potential strategy for the treatment of *H. pylori*-associated gastric carcinoma.
